# Trends and correlates of HIV-1 resistance among subjects failing an antiretroviral treatment over the 2003–2012 decade in Italy

**DOI:** 10.1186/1471-2334-14-398

**Published:** 2014-07-18

**Authors:** Marco Franzetti, Michela Violin, Andrea Antinori, Andrea De Luca, Francesca Ceccherini-Silberstein, Nicola Gianotti, Carlo Torti, Stefano Bonora, Maurizio Zazzi, Claudia Balotta

**Affiliations:** 1 Department of Biomedical and Clinical Sciences 'L. Sacco', Infectious Diseases and Immunopathology Section, University of Milan, Milan, Italy; 2 National Institute for Infectious Diseases ‘L. Spallanzani’ IRCCS, Roma, Italy; 3 Infectious Diseases Unit, Siena University Hospital, Siena, Italy; 4 Department of Experimental Medicine, University of Rome Tor Vergata, Roma, Italy; 5 Infectious Diseases, San Raffaele Scientific Institute, Milan, Italy; 6 University Department of Infectious Diseases, University of Brescia, Brescia, Italy; 7 Unit of Infectious Diseases, Department of Medical and Surgical Sciences, University “Magna Graecia”, Catanzaro, Italy; 8 University of Turin, Turin, Italy; 9 Department of Molecular Biology, University of Siena, Siena, Italy

**Keywords:** HIV, Antiretroviral therapy, HIV acquired resistance

## Abstract

**Background:**

Despite a substantial reduction in virological failures following introduction of new potent antiretroviral therapies in the latest years, drug resistance remains a limitation for the control of HIV-1 infection. We evaluated trends and correlates of resistance in treatment failing patients in a comprehensive database over a time period of relevant changes in prescription attitudes and treatment guidelines.

**Methods:**

We analyzed 6,796 HIV-1 *pol* sequences from 49 centres stored in the Italian ARCA database during the 2003–2012 period. Patients (n = 5,246) with viremia > 200 copies/mL received a genotypic test while on treatment. Mutations were identified from IAS-USA 2013 tables. Class resistance was evaluated according to antiretroviral regimens in use at failure. Time trends and correlates of resistance were analyzed by Cochran-Armitage test and logistic regression models.

**Results:**

The use of NRTI backbone regimens slightly decreased from 99.7% in 2003–2004 to 97.4% in 2010–2012. NNRTI-based combinations dropped from 46.7% to 24.1%. PI-containing regimens rose from 56.6% to 81.7%, with an increase of boosted PI from 36.5% to 68.9% overtime. In the same reference periods, Resistance to NRTIs, NNRTIs and PIs declined from 79.1% to 40.8%, from 77.8% to 53.8% and from 59.8% to 18.9%, respectively (p < .0001 for all comparisons). Dual NRTI + NNRTI and NRTI + PI resistance decreased from 56.4% to 33.3% and from 36.1% to 10.5%, respectively. Reduced risk of resistance over time periods was confirmed by a multivariate analysis.

**Conclusions:**

Mutations associated with NRTIs, NNRTIs and PIs at treatment failure declined overtime regardless of specific class combinations and epidemiological characteristics of treated population. This is likely due to the improvement of HIV treatment, including both last generation drug combinations and prescription guidelines.

## Background

Despite substantial reductions in AIDS-related morbidity and mortality [1,2], advancements in antiretroviral therapy have always been challenged by the development of drug resistance. Evolution of drug-resistant HIV variants remains a major limitation for the long-term control of HIV-1 infection as wide resistance to multiple drug classes is associated with clinical deterioration and death [3]. A multitude of drug-resistant strains have been reported that differ considerably in their susceptibility to the three major classes of antiretrovirals in use since the introduction of the highly active aniretroviral treatment (HAART): nucleos(t)ide reverse-transcriptase inhibitors (NRTIs), non-nucleoside reverse-transcriptase inhibitors (NNRTIs) and protease inhibitors (PIs) [4]. The prevalence of resistant strains rose to high levels in high-income countries in the past, when treatment often made of suboptimal regimens became available [5-8]. Drug resistance has emerged recently also in low/middle-income countries, as a consequence of the growing access to first- generation drug combinations coupled with the high burden of HIV infection [9,10].

Treatment failing patients appear to be the major cause of the transmission of resistant strains [11] which may compromise the efficacy of combination antiretroviral treatment (cART) in drug-naive patients [12]. Nevertheless, when the initial cART is tailored to the viral genotype to ensure full activity, patients can achieve virological responses comparable to those harbouring wild type virus [13]. Therefore, it is expected that current optimal regimens, selected on the basis of HIV *pol* genotype, control viral replication even in patients with primary resistance.

Available results on the prevalence of drug resistance are often difficult to compare as they differ in resistance associated mutations taken into account, timing of samples and selection of study populations. The latter could vary from all subjects on antiretroviral treatment, to patients failing ART, to subjects with available resistance test results. Moreover, these studies have estimated the prevalence of HIV drug resistance using a variety of analytical methods resulting in a wide range of estimates, ranging from 50% to 80% of subjects failing an antiretroviral treatment [5-8,14-16].

A reduction of acquired resistance has been recently reported in Italy until 2009 [17,18]. This may have derived from the latest prescription attitudes and, likely, to the introduction of more potent new drugs in salvage therapies. Nevertheless, considerable proportions of treated individuals are still likely to select for resistance mutations while on antiretroviral treatment, which may result in an ongoing transmission of HIV-1 resistant variants.

The aim of this study was to monitor acquired resistance to understand present trends and correlates of class resistance in subjects failing cART regimens in a multicenter Italian network based over the 2003–2012 period. Previous reports considered resistance prevalence among the whole studied population, regardless of the regimen administered at the time of resistance testing [16-18]. Since treatment changes can influence drug pressure on previously selected virus variants, we chose instead to evaluate resistance trends according to the antiretroviral drugs taken at time of failure.

## Patients and methods

### Patients

Patients included in the study were adult HIV-infected individuals enrolled in 49 Italian clinical centres during the 2003–2012 period. All the clinical centres contributed data to the Antiretroviral Resistance Cohort Analysis (ARCA, http://www.hivarca.net) database, a nationwide repository used for non-profit research purposes and stored on a central server. Written informed consents had been obtained by patients. The research did not require approval from the Ethics Committees, according to the Italian law at the time when the study was conducted, since it was performed as an observational study in the context of clinical routines (art.1, Low. Decree 211/2003).

### Inclusion criteria

Cases were selected according to DHHS Guidelines [19] on the basis of the concomitant detection of HIV-1 viral load over 200 copies/ml after at least 6 months of ongoing therapy and the availability of an HIV-1 genotypic test obtained while on treatment. The cART regimen was defined as any combination of three or more drugs including an NNRTI and/or a PI. When more than one sequence was available from the same subject in the same year of study, the first sequence was considered.

### HIV-1 genotype and class resistance evaluation

Genotyping was based on a partial HIV-1 *pol* sequence including RT and protease and ranging from 1,000 to 1,280 nucleotides, depending on the sequencing protocol used at the contributing laboratory. Emergence of resistance at failure was evaluated according to the latest International AIDS Society (IAS) mutation list [20]. Any thymidine analogue mutations (TAMs) (M41L, D67N, K70R, L210W, T215Y/F and K219Q/E), K65R, L74I/V, Y115F, Q151M, 69ins, M184I/V, any major NNRTI mutation (L100I, K101P, K103N/S, V106A/M, V108I, Y181C/I, Y188C/H/L, G190A/S, P225H, M230L) and the presence of major PI mutations (D30N, V32I, L33F, M46I/L, I47A/V, G48V, I50L/V, I54L/M, Q58E, L76V, V82A/F/L/S/T, I84V, N88D/S, L90M) were considered. TAMs were classified according to different patterns in profile 1 (M41L, L210W and T215Y) and profile 2 (D67N, K70R, T215F, K219Q/E). Specifically, only mutations related to drug in use at genotype were taken into account.

NRTI, NNRTI and PI class resistance was evaluated according to NRTI, NNRTI or PI based treatment used at time of virological failure.

Four periods of study were evaluated encompassing years 2003–2004, 2005–2006, 2007–2009 and 2010–2012.

Subtype was assigned using the NCBI HIV-1 subtyping tool.

### Statistical analysis

The Cochrane-Armitage test was used to evaluate temporal trends. The crude and Mantel-Haenszel adjusted odds ratios (OR) of class resistance detection with 95% confidence interval (CI) were calculated. Univariate analysis was performed using *χ*^*2*^ and logistic regression. A subsequent multivariate analysis was done on all variables, using the same tests with a full model. Analyses were done with SAS Software version 9.1.

## Results

### Characteristics of the population

The sequences were obtained from 5,246 HIV-1 positive individuals. Overall, male to female ratio was 2.1 (3,554/1,692), the route of transmission was injection drug use (IDU) for 35.4% (n = 1,858), heterosexual sex in 33.5% (n = 1,757), and men having sex with men (MSM) were 16.7% (n = 876) of the study population. Non-B subtypes were detected in 9.1% of the subjects included in the study (n = 477). For 4,130 (78.7%) patients a single *pol* sequence was available for the analysis. Two, 3, 4 and more than 4 sequences were collected for 800, 227, 65, 24 subjects (15.2%, 4.3%, 1.2%, 0.5% respectively).

The study included 6,796 *pol* sequences from subjects with viral load above 200 copies/ml while on treatment in the 2003–2012 period. Their distribution was as follows: 2,450 in 2003–2004 (36.1%); 2,050 in 2005–2006 (30.2%); 1,641 in 2007–2009 (24.2%); 655 in 2010–2012 (9.6%). Median age of patients at time of genotyping was 42 years (Interquartile ratio, IQR 38–47). Median HIV-RNA viral load was 4.06 Log copies/ml (IQR 3.36-4.74) and median CD4 number was 282 cells/μl (IQR 168–429 cells/μl). The median number of previous treatments experienced by enrolled subjects was 5 for each patient (IQR 2–9).

### Patients’ treatment at time of genotypic test

At genotypic testing 99.4% (n = 6,757), 37.4% (n = 2,539) and 67.5% (n = 4,589) sequences were obtained from NRTI-, NNRTI- or PI-treated patients, respectively. HAART combination included NRTIs + NNRTI in 32.5% (n = 2,207), NRTIs + PI(s) in 62.6% (n = 4,257), NRTI(s) + PI + NNRTI in 4.3% (n = 293), an NNRTI + PI(s) in 0.6% (n = 39).

**Figure 1:**
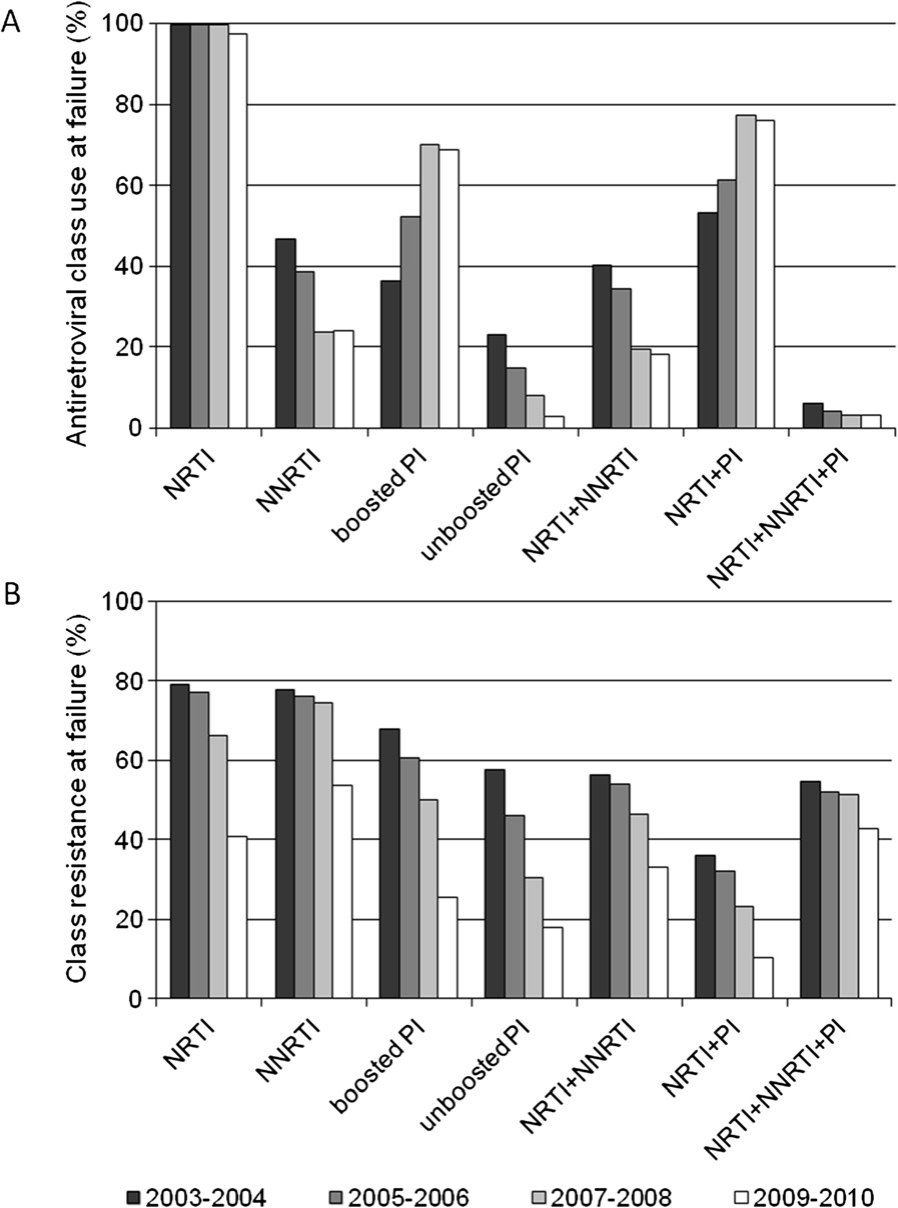
**Trends in drug class usage and resistance at failure. Part A:** Distribution of antiretroviral classes in use at time of genotyping when HIV-RNA > 1 Log copies/ml: trends of use are shown for nucleoside reverse-transcriptase inhibitors (NRTI, p < .0001*), non nucleoside reverse-transcriptase inhibitors (NNRTI, p < .0001*), protease inhibitors (PI, p < .0001*), Boosted PI (p < .0001*), antiretroviral regimens combining NRTI + NNRTI (p < .0001*), NRTI + PI (p < .0001*), NRTI + NNRTI + PI (p < .0001*). **Part B:** Prevalence of drug class resistance during the study period: trends of resistance are shown for NRTI (p < .0001*), NNRTI (p < .0001*), PI (p < .0001*), Boosted PI (p < .0001*), antiretroviral regimens combining NRTI + NNRTI (p < .0001*), NRTI + PI (p < .0001*), NRTI + NNRTI + PI (n.s.*). * Cochrane-Armitage test for trends.

NRTI usage significantly decreased overtime, ranging from 99.7% in 2003–2004 to 97.4% in 2010–2012 (p < .0001). NNRTI-based regimens decreased from 46.7% to 24.1% (p < .0001), while an increase of cART regimens including PIs was observed (from 59.6%, to 81.7%, p < .0001) overtime. The same increasing trend was confirmed for ritonavir boosted PI regimens (from 36.5%, to 68.9%, p < .0001).Antiretroviral classes were administered in several combinations during the study period: the prevalence of the association of NRTIs + NNRTI decreased from 40.4% to 18.3% (p < .0001), while combinations of NRTIs + PI(s) increased from 53.4%, to 75.9% (p < .0001). The combination of all the three classes showed a significant declining trend from 6.0% to 3.2% (p < .0001) (Figure 1, Part A). The association between an NNRTI and a PI(s) increased significantly from 0.3% to 2.6% (p < .0001) in the study period.

Substantial variations were found in the prevalence of use of some single NRTI agents at failure. A decrease was observed for thymidine analogues (TA), didanosine (ddI) and lamivudine (3TC); their use went from 63.0% to 16.0% (p < .0001), from 36.6% to 1.7% (p < .0001) and from 63.0%, to 35.6%, (p < .0001), respectively. An opposite trend was observed for tenofovir (TDF) from 28.7%, to 63.4% (p < .0001), abacavir (ABC) from 8.9% to 16.6% (p < .0001) and emtricitabine (FTC) from 0% to 57.7% (p < .0001). Regarding NNRTIs, a decrease of both nevirapine (NVP) and efavirenz (EFV) use was detected at failure; their use went from 24.1% to 7.0% (p < .0001) and from 22.6% to 12.5% (p < .0001), respectively. Among subjects assuming an NNRTI, the rate of subjects in treatment regimens including NVP decreased from 51.6% to 29.1% (p < .0001). Among PIs, significant reductions in usage frequency at failure were observed for indinavir (from 6.2% to 0.3%, p < .0001), nelfinavir (from 17.7% to 0.0%, p < .0001), and saquinavir (from 6.2% to 1.7%, p < .0001). In contrast, an increasing trend was observed for lopinavir (from 25.0% to 28.1%, p = .012), fosamprenavir (from 0% to 7.0%, p < .0001), atazanavir (from 2.3% to 23.8%, p < .0001), darunavir (from 0% to 16.3%, p < .0001), and tipranavir (from 0.6% to 1.6%, p < .0001).

### Trends of drug resistance at failure

Resistance to NRTIs declined from 79.1% to 40.8%, from 2003–2004 to 2010–2012 (p < .0001). A decreasing trend was also found for NNRTI mutations, which dropped from 77.8% to 53.8% (p < .0001). PI mutations decreased from 59.8% to 18.9% (p < .0001). Among subjects assuming NRTIs combined with an NNRTI, resistance to both classes went from 56.4% to 33.3% (p < .0001); resistance to NRTI + PI went from 36.1% to 10.5% (p < .0001). Triple class resistance did not significantly vary overtime in subjects assuming NRTI(s) + NNRTI + PI, ranging from 54.8% to 42.9% in the study period (Figure 1, Part B).

Declining rates of TA-mutations were found in TA-containing regimens from 55.3% (853/1543) to 7.62% (8/105) (p = <.0001). A significant decline was observed for both TAM1 and TAM2 profiles, ranging from 30.4% (469/1543) to 3.8% (4/105) (p < .0001) and from 19.6% (302/1543) to 1.9% (2/105) (p < .0001), respectively. The K65R mutation went from 4.1% (101/2438) to 2.2% (14/637) (p = 0.0071) in regimens based on TDF, ddI, stavudine and/or ABC. The M184I/V mutations dropped from 67.3% (1131/1680) to 34.1% (211/618) (p < .0001) in patients on 3TC-, FTC,- or ABC-containing regimens. This mutation was more common in subjects assuming 3TC rather than FTC (61.3% vs. 41.6%, p = 0.001). Among subjects failing ABC- or ddI-including cART, the L74V mutation decreased from 16.5% (165/1000) to 3.3% (4/120) (p < .0001).

Frequencies of specific mutations and resistance patterns are shown in Table 1 for NNRTI-and PI- based cART. Among NNRTI-based treatments, a significant decline was found for K103N/S, V106A/M, V108I, Y181C/I and G190S/A. No significant variations were detected for L100I, 188C/L/H, P225H and M230L. In PI-containing regimens, a reduction of frequency was found for D30N, L33F, M46I/L, G48V, L76V, V82A/F/T/S, I84V, N88D/S and L90M. A significant increase was detected for I50L and I54L/M. No significant variation was found for V32I, I47A/V, I50V and Q58E.

**Table 1:** Frequencies of major non nucleoside reverse-transcriptase inhibitor (NNRTI) and protease inhibitor (PI)

**NNRTI**^**a**^**-mutations**					
**Mutations**	**2003-2004 (n = 1,143) % (n)**	**2005-2006 (n = 799) % (n)**	**2007-2009 (n = 439) % (n)**	**2010-2012 (n = 158) % (n)**	***P***^***c***^
L100I	6.65 (76)	8.01 (64)	5.92 (26)	8.86 (14)	ns
K103N/S	45.83 (525)	43.30 (346)	31.66 (139)	32.28 (51)	<.0001
V106A/M	6.47 (74)	4.51 (36)	3.64 (16)	3.80 (6)	.0122
V108I	11.64 (133)	10.64 (85)	10.25 (45)	5.70 (9)	.0476
Y181C/I	22.83 (261)	21.78 (174)	15.26 (67)	15.19 (24)	.0005
Y188C/H/L	5.34 (61)	6.01 (48)	5.24 (23)	6.96 (11)	ns
G190A/S	22.66 (259)	20.78 (166)	13.44 (59)	12.03 (19)	<.0001
P225H	5.07 (58)	5.26 (42)	4.10 (18)	4.43 (7)	ns
M230L	1.57 (18)	1.38 (11)	1.82 (8)	0.63 (1)	ns
**PI**^**b**^**-mutations,% (n)**					
**Mutations**	**2003-2004 (n = 1,461) % (n)**	**2005-2006 (n = 1,331) % (n)**	**2007-2009 (n = 1,262) % (n)**	**2010-2012 (n = 535) % (n)**	***P***^***c***^
D30N	9.5 (139)	5.9 (79)	2.0 (25)	0.9 (5)	<0.0001
V32I	4.8 (70)	5.0 (66)	6.9 (86)	4.1 (22)	ns
L33F	13.1 (191)	16.1 (214)	15.5 (196)	6.0 (32)	<0.0001
M46I/L	32.6 (477)	32.0 (426)	25.6 (323)	12.1 (65)	<0.0001
I47A/V	5.5 (81)	5.1 (68)	5.6 (71)	2.4 (13)	ns
G48V	5.3 (77)	4.1 (54)	1.4 (18)	0.4 (2)	<0.0001
I50L	0.5 (7)	1.8 (24)	1.9 (24)	1.7 (9)	0.004
I50V	2.0 (29)	2.0 (26)	1.9 (24)	0.9 (5)	ns
I54L/M	4.5 (66)	5.9 (78)	9.2 (116)	5.2 (28)	0.010
Q58E	5.9 (87)	9.0 (120)	8.1 (102)	4.9 (26)	ns
L76V	3.7 (54)	3.5 (47)	3.2 (40)	1.3 (7)	0.020
V82A/F/T/S	26.9 (393)	27.1 (361)	17.7 (224)	6.4 (34)	<0.0001
I84V	13.5 (198)	14.9 (198)	16.0 (202)	5.4 (29)	0.015
N88D/S	8.1 (119)	6.8 (90)	3.4 (43)	2,2 (12)	<0.0001
L90M	31.5 (461)	29.8 (397)	20.8 (262)	9.5 (51)	<0.0001

NNRTI and PI resistance mutations were evaluated among subjects failing a NNRTI- or a PI-containing regimen, respectively.

^a^Non nucleoside reverse-transcriptase inhibitor.

^b^Protease inhibitor.

^c^Cochrane-Armitage test for trends.

ns: not significant.

### Predictors of class resistance

Table 2 shows the uni- and multivariate logistic regression analysis investigating possible predictors of class resistance development on treatment. NRTI resistance in patients assuming any drug of this antiretroviral class was found in association with several independent predictors, including gender, risk factor, age, HIV-1 subtype, viral load at failure, previous virological failures, number of previous antiretroviral regimens, prior exposure to suboptimal NRTI therapy, associated antiretroviral class in use at failure and period of study.

**Table 2:** Predictors of nucleoside reverse-transcriptase inhibitor (NRTI), non-nucleoside reverse-transcriptase inhibitor (NNRTI) and protease inhibitor (PI) resistance

	**NRTI**^**a **^**resistance OR (95% CI)**		**NNRTI**^**b **^**resistance OR (95% CI)**		**PI**^**c **^**resistance OR (95% CI)**	
	**Univariate**	**Multivariate**	**Univariate**	**Multivariate**	**Univariate**	**Multivariate**
**Gender,** Female vs. Male	0.74 (0.66-0.82)	0.67 (0.58-0.77)	0.93 (0.77-1.11)	0.82 (0.68-0.98)	0.62 (0.55-0.70)	0.59 (0.50-0.69)
**Risk factor**						
**Intravenous drug use**	-	-	-	-	-	-
**Men having sex with men**	1.17 (0.99-1.38)	1.26 (1.05-1.51)	0.63 (0.49-0.81)	0.84 (0.70-1.03)	1.47 (1.22-1.76)	1.45 (1.18-1.79)
**Heterosexual sex**	1.01 (0.89-1.14)	1.58 (1.36-1.84)	0.78 (0.63-0.97)	0.94 (0.72-1.27)	1.01 (0.88-1.17)	1.61 (1.36-1.91)
**Other**^**d**^	0.87 (0.75-1.01)	1.68 (1.40-2.01)	0.61 (0.47-0.79)	0.89 (0.64-1.23)	0.89 (0.75-1.06)	1.53 (1.26-1.87)
**Age,** per 10 years older	1.17 (1.10-1.25)	1.09 (1.01-1.17)	0.93 (0.84-1.03)	0.96 (0.85-1.09)	1.17 (1.09-1.25)	1.05 (0.97-1.14)
**HIV-1 Subtype, Non-B vs. B**	0.48 (0.40-0.56)	0.79 (0.65-0.97)	0.58 (0.44-0.77)	0.80 (0.56-1.15)	0.48 (0.38-.59)	0.83 (0.64-1.06)
**Viral load**						
**> 5 Log copies/ml**	-	-	-	-	-	-
**4 - 5 Log copies/ml**	2.36 (2.04-2.73)	2.16 (1.83-2.55)	2.01 (1.56-2.58)	1.71 (1.23-2.35)	1.83 (1.54-2.17)	1.68 (1.38-2.04)
**< 4 Log copies/ml**	2.92 (2.54-3.36)	3.06 (2.59-3.61)	3.22 (2.51-4.13)	2.61 (1.89-3.60)	1.60 (1.36-1.89)	1.70 (1.40-2.07)
**CD4 cell count**						
**>350 cells/mmc**	-	-	-	-	-	-
**200 – 350 cells/mmc**	0.65 (0.58-0.74)	0.90 (0.78-1.03)	1.04 (0.85-1.27)	1.07 (0.84-1.36)	0.90 (0.78-1.04)	0.86 (0.73-1.01)
**< 200 cells/mmc**	0.82 (0.72-0.93)	0.87 (0.75-1.01)	1.01 (0.81-1.25)	1.36 (1.02-1.80)	0.83 (0.72-0.96)	0.88 (0.66-1.12)
**Previous virological failure(s)**	4.41 (3.84-5.07)	2.07 (1.74-2.47)	4.52 (3.63-5.63)	2.95 (2.17-3.99)	3.88 (3.17-4.75)	1.40 (1.10-1.78)
**Prior ARV regimens,** per 1 higher	1.16 (1.15-1.18)	1.11 (1.09-1.13)	1.14 (1.11-1.17)	1.03 (0.99-1.07)	1.21 (1.18-1.24)	1.13 (1.11-1.15)
**Prior suboptimal NRTI therapy**	3.45 (2.85-4.17)	1.50 (1.29-1.74)	2.52 (2.11-3.00)	1.04 (0.80-1.35)	3.16 (2.80-3.57)	1.27 (1.07-1.50)
**Antiretrovira regimen in use**			-	-	-	-
**NNRTI + NRTIs**	-	-				
**Boosted PI + NRTIs**	0.95 (0.85-1.06)	0.65 (0.56-0.74)				
**Unboosted PI + NRTIs**	0.84 (0.73-0.98)	0.75 (0.63-0.90)				
**PI + NNRTI + NRTI**	3.51 (2.66-4.65)	1.87 (1.25-2.78)				
**Boosted vs unboosted PI**	-	-	-	-	1.00 (0.88-1.15)	1.06 (0.90-1.23)
**Study period,** per 1 period higher	0.56 (0.53-0.59)	0.58 (0.54-0.61)	0.65 (0.59-0.71)	0.74 (0.66-0.82)	0.58 (0.55-0.62)	0.58 (0.54-0.62)

A logistic regression model was performed in subjects failing a combined antiretroviral treatment according to antiretrovirals in use at genotype.

^a^Nucleoside reverse-transcriptase inhibitors.

^b^Non nucleoside reverse-transcriptase inhibitors.

^c^Protease inhibitors.

^d^Other: professional risk, transfusions¸ vertical transmission.

Independent predictors of NNRTI resistance in NNRTI-assuming subjects were gender, viral load at failure, previous virological failures.

Predictors of PI resistance were gender, risk factor, viral load at failure, previous virological failures, number of previous antiretroviral regimens, prior exposure to suboptimal NRTI therapy, use of TA and period of study. The usage of boosted rather than unboosted PIs was not associated with the risk of evolution on PI resistance.

The risk of resistance to any antiretroviral class was not influenced by the use of TA-including or sparing regimens or by the use of FTC rather than 3TC in the multivariate model, even though a lower prevalence of any NNRTI resistance was found in subjects assuming FTC compared to 3TC (52.6% vs. 61.2%, p = 0.012).

## Discussion

Our data provide a solid evidence of a marked decrease in the prevalence of class resistance among treated individuals in Italy, as observed from a retrospective analysis including 49 clinical centres participating in the ARCA cohort during the last decade. Studies exploring resistance trends in European countries have observed an initial decrease over the 2005–2008 period [15-18,21]. However, HIV resistance to antiretrovirals still deserves continuous monitoring, extending the observation to more recent years and providing updated understanding of the correlates of resistance.

Differently from previous works [14,15] the design of our study focused on HIV drug resistance in patients assuming the corresponding drugs at the time of failure, providing a more accurate estimate with respect to using the whole pre-treated population [16-18]. This approach was adopted mainly to correct for the large observed variation in antiretroviral usage over time, specifically taking into account the antiretroviral regimen in use at failure.

This analysis allowed to detect a twofold and threefold decrease in resistance prevalence to NRTIs and PIs, respectively during the 2003–2012 decade. The study periods included two biennia and two triennia to balance the distribution of subjects overtime. NNRTI resistance declined less markedly, from 77.8% to 53.8%. Nevertheless, the reduction of NNRTI resistance overtime is more relevant than that observed in previous studies [18,22] even though a rapid selection of resistant variants is observed in patients failing NNRTI based regimens [23]. We observed an important reduction of specific NRTI mutations, such as K65R, L74V, M184V and TAMs in subjects administered drugs selecting for these mutations. This decrease may be due to multiple factors including the introduction of compact dual NRTI formulations and association with high genetic barrier boosted PIs. Several NNRTI mutations did not significantly vary overtime: a stable rate of selection was observed for L100I, 188C/L/H, P225H and M230L. A declining prevalence of K103N/S and Y181C/I overtime may be explained by the reduced usage of EFV and NVP which preferentially select for such mutations [24]. A similar declining trend can be observed when exploring the variations of specific PI mutations, which are largely influenced by the different variations in use of specific PIs overtime. For example, we observed an increase of I50L and I54L/M, which have been reported to be major mutations for atazanavir and darunavir, respectively [25].

Several epidemiological correlates were found to have an impact over the selection of resistance mutations only for NRTIs and PIs. An increased risk of resistance was found for sexual routes of transmission (heterosexual and homosexual) compared to IDU, in agreement with other Italian studies [17,18]. This finding may be explained by different adherence patterns in these subsets of patients as the higher rates of resistance among subjects who acquired the infection through sexual route may be due to suboptimal, even though intermediate to high, levels of adherence in this population. By contrast, among IDUs, very low levels of compliance to medical prescriptions can lead to an important reduction of drug levels, which may not result in sufficient drug selective pressure leading to resistance to NRTIs or PIs [22]. We also observed a higher risk of resistance to each class of antiretrovirals among males rather than females. This association with gender should take into account a possible lower adherence observed in female subjects [26,27].

Lower drug resistance rates were associated with plasma HIV-1 viremia above 100,000 copies/ml. This finding is different from previous observations obtained from the ARCA database [17] and it is in agreement with findings in patients undergoing resistance testing in routine clinical practice [28]. This may be due to cases of very low adherence, when low drug levels reduce both the efficacy of antiretroviral therapy and emergence of resistant strains [27]. Our observation underlines the need of genotypic testing at the very early detection of virological failure, when the HIV-1 viremia is still at a low level, as suggested by recent guidelines [19].

As expected, the occurrence of a previous virological failure increased the risk of resistance to any antiretroviral class. Other treatment history features, such as the number of cART regimens, have shown a role in the emergence of class resistance to NRTIs and PIs, but not to NNRTIs. The fact that NNRTI resistance shown no association with the temporal length of antiretroviral treatment can be explained by the characteristic pattern of resistance evolution of this antiretroviral class, whose use can be compromised by a single substitution leading to cross resistance [29].

The risk of resistance to NRTIs was higher when the patients failed NNRTI rather than PI based treatments. This is in agreement with previous findings and confirms that a higher protection against NRTI resistance is obtained by cART regimens including antiretrovirals with a higher genetic barrier [17,18,20]. However, this protection against the emergence of NRTI resistance was observed either for boosted or unboosted PIs compared to NNRTIs when the entire time period of the study was considered. Similarly, no difference in risk of PI mutations evolution could be found when comparing boosted and unboosted PIs overtime. Enven though unexpected, these observations are in agreement with data demonstrating that comparable virological outcome was achieved when the same PI, boosted or unboosted, was used in antiretroviral regimens in specific clinical settings and when allowed by guidelines [30,31].

Interestingly, M184V and NNRTI resistance were less common in subjects failing regimens including emtricitabine rather than lamivudine, as observed by previous studies [32]. This finding may be also partly related to the availability and increasing use of fixed dose combinations containing FTC, which have been shown to implement adherence [33,34]. Nevertheless, no significant impact on NNRTI resistance was observed when considering the concomitant use of specific NRTI backbones (3TC vs. FTC) or NNRTIs (EFV vs. FTC) in the multivariate model. Of note, TA-sparing regimens did not appear to decrease the risk of resistance. Even though the virological efficacy of TAs is generally confirmed in several clinical settings [35], our finding does not reduce the usefulness and virological efficacy of TA sparing compounds. In fact, our data show a continuous drop in resistance prevalence in the Italian HIV-infected population during a period when TA-sparing regimens substantially substitute TA-including combinations, mainly due to toxicity issues.

Some limitations of our work should be acknowledged. First of all, Italian data on precise extent of HIV-1- treated population, specific treatment prescriptions and related overall achievement of virological suppression on cART are not available at present. In fact, an increase of treatment effectiveness could explain both the reduction of the available resistance testing and the extent of resistance to antiretrovirals. Moreover, we were not able to consider the impact of self-reported adherence in our study population, due to the lack of information regarding this issue for a large number of patients, referring to several clinical centers in Italy. Furthermore, patients with resistant strains in early years of the study could have been lost at follow-up, due to several reasons including death, contributing to the decline of overall resistance. As guidelines prescribe to perform a genotypic resistance testing at failure, almost all patients failing an antiretroviral regimens have been included in the analysis. Nevertheless, the full information about the number of subjects failing antiretroviral regimens is not available, and we cannot exclude a partial loss of information regarding subjects who were not tested for antiretroviral resistance.

Even though it was not possible to evaluate all the data regarding the antiretroviral history of patients, our analysis included main covariates such as previous virological failures, number of previous antiretroviral regimens, prior exposure to suboptimal NRTI therapy. Importantly, this work includes an evaluation of resistance according to the antiretroviral regimen in use at failure, representing an achievement in the analysis of acquired resistance not performed in previous works [17,18,21].

In spite of the limitations mainly due to its retrospective and multicenter design, our study shows a substantial decrease in drug resistance after stratification for antiretroviral class in use at failure. This finding is encouraging for the goal of a more effective treatment of the HIV infected population, in order to achieve a stable reduction of the potential virus transmitters. Moreover, the reduction we observed in resistance prevalence among treated individuals is probably the main driving factor leading to the observed reduction in primary resistance in our country [36].

## Conclusion

In conclusion, our analysis shows a reduction of drug resistance overtime, even when correcting for several other or covariates or confounders. This is likely the consequence of time-associated factors including the availability of new potent drugs, updated guidelines for the treatment of HIV infection, the availability and frequent use of resistance testing to select for the optimal antiretroviral treatment at baseline and whenever a virological failure occurs.

## Competing interests

A.D.L. has received funds for research from ViiV and he is a member of the Gilead speakers bureau and Siemens Diagnostics, ViiV, Gilead, Janssen-Cilag and Abbvie Advisory boards. He also received travel grants from Bristol-Myers Squibb and ViiV. F. C-S. has received funds for attending symposia, speaking, organizing educational activities, grant research support, consultancy and advisory board from Abbott, Merck Sharp & Dohme, Gilead, Janssen Cilag, Roche, Bristol Myers Squibb, and ViiV. M.Z. has received research funds from ViiV, has received speaker fees from Merck Sharp and Dohme, Janssen-Cilag and Gilead, has been a memeber of the advisory board of Abbott Molecular.

## Authors’ contributions

MF, MV, MZ and CB participated in the design of the study. MF and MV drafted the manuscript. MV performed the statistical analysis. AA, ADL, FCS, NG, CT and SB provided the clinical and virological data stored in the ARCA database. MZ and CB coordinated the study and supervised the manuscript. All authors read and approved the final manuscript.

## Pre-publication history

The pre-publication history for this paper can be accessed here:

http://www.biomedcentral.com/1471-2334/14/398/prepub
